# A report on case reports

**DOI:** 10.4103/0972-0707.73375

**Published:** 2010

**Authors:** Velayutham Gopikrishna

**Affiliations:** Journal of Conservative Dentistry Editor (2008-2010), Department of Conservative Dentistry and Endodontics, Thai Moogambigai Dental College, Chennai 600 107, India

**Keywords:** Case report, dental writing, publishing

## Abstract

Case reports are defined as the scientific documentation of a single clinical observation and have a time-honored and rich tradition in medicine and scientific publication. This article discusses the role and relevance of case reports in the current evidence-based medical literature. It also seeks to help and guide authors to understand how to prepare a reasonable and well-written case report and how they may anticipate concerns that peer reviewers may express when scrutinizing their manuscript. An overview of the *Journal of Conservative Dentistry’s* review process of a manuscript submission is provided for the benefit of future authors. It is important to be able to read a case report critically and to use the information they contain appropriately. This article also discusses the factors to consider in evaluating individual case reports, and discusses a practical conceptual scheme for evaluating the potential value and educational content of a case report.

## INTRODUCTION

Always note and record the unusual…Publish it. Place it on permanent record as a short, concise note. Such communications are always of value.

– Sir William Osler

The clinical case report, which describes and analyzes the diagnosis and/or the management of one or two patients, is the first line of evidence in health care.[1–3] A case report is a powerful tool to disseminate information on unusual clinical syndromes, disease associations, unusual side effects to therapy, or response to treatment. Case reports have been used for years as a means to teach health sciences students,[2,4] and are one of the best ways for authors to get started in scholarly writing,[2,5,6] and can be a valuable learning experience for both author and reader. Case reports continue to be a very popular section within the Journal. They are well read, and by nature they are easily accessible.[6]

**Figure 1:**
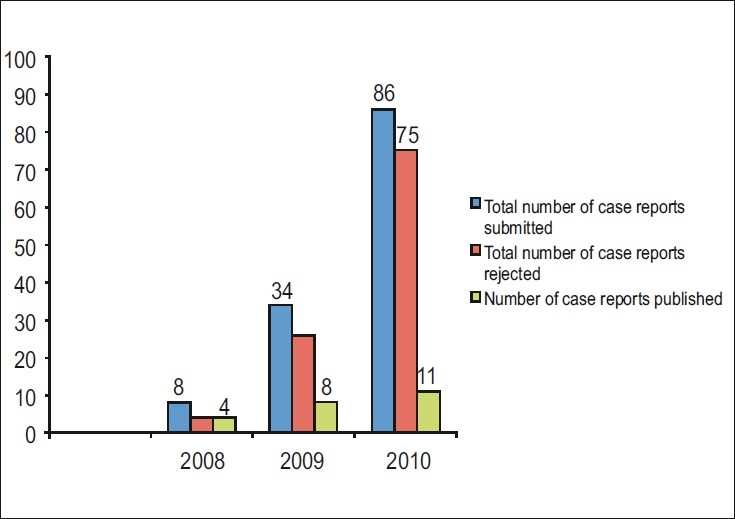
The number of manuscripts that are submitted as a case report to the *Journal of Conservative Dentistry* in comparison with the number of manuscripts that were accepted for publication and those that were rejected for the period 2008–2010.

Among the many reasons that explain the popularity of case reports, the main one is probably the accessible nature of this particular piece of clinical information. Symbol of the high popularity in the Journal is the continuous rise in the number of case report submissions [Figure 1]. The Journal has always witnessed a steady stream of case reports, but over the last years the number has increased significantly. This year we received 86 case reports, and this represented a *tenfold * increase in comparison with 2008.

The *Journal of Conservative Dentistry* receives more case reports than it can publish. Unfortunately, although many of these manuscripts are academically worthy, they are too poorly written to merit acceptance. Some manuscripts are considered beyond salvage, and these are rejected outright. Other manuscripts pass through one or more rounds of peer review before an editorial decision is taken. In both these situations, and especially in the latter, the editorial office and the reviewers of the manuscripts are taxed, this wastes time and resources, and the waste is a complete loss for all involved if the manuscript is eventually rejected.

This article seeks to help and guide authors to understand how to prepare a reasonable and well-written case report. Writing a good research paper is an art that requires skills in both academic and literary domains. Articles that are better in quality at the time of original submission will stand a higher chance of acceptance.

This article hopes to help authors to better understand the nuances involved in the preparation of a case report and how they may anticipate concerns that peer reviewers may express when scrutinizing their manuscript. The objectives of this article are to improve the chances that authors will receive a favorable review for their own manuscripts, and to thereby reduce the burden that the reviewers and editorial office of the *Journal of Conservative Dentistry* experience with submissions that require extensive and repeated rounds of revision.

## THE RELEVANCE OF CASE REPORTS IN CONTEMPORARY JOURNALS

Case reports constitute a small segment of the medical literature; about 7% of the articles published in general medical and family practice journals, according to one study.[7] The single case report occupies a pretty low rung on the ladder of evidence-based medicine, which today’s students, investigators, and clinicians are admonished to climb diligently in their quest for scientific truth and rational clinical decision making. Available schemes for ranking the various levels of evidence place randomized controlled trials at the top – superseded only by meta-analyses of multiple randomized controlled trials and retrospective studies, case series, and unsystematic observations at the bottom.[8] Most such hierarchies do not list case reports at all. Those that do, relegate them to the evidence ladder’s lowest rungs along with the anecdotal observation and expert opinion. Data from a single case, and any conclusions or speculation drawn from it, clearly do not have the weight of findings of the other types of research studies such as clinical trials,[9] retrospective studies,[10] and surveys.[11]

On the other hand, several authors have pointed out that, prepared carefully and interpreted with appropriate circumspection, case reports have a valuable part to play in both the advance of medical knowledge and the pursuit of education.[12–15]

A well-documented account of something not previously reported in the literature can be a useful contribution. In his monograph on writing and publishing in the health sciences, Huth[16] lists four types of cases that may constitute worthwhile contributions to the medical literature:

- A unique case that may represent a previously unknown syndrome or disease.
- A case with the previously unreported association of two distinct diseases, suggesting a possible relationship between them.
- An “outlier” with features strikingly outside the realm of what is usually seen with a particular disease.
- An unexpected response or course suggesting a previously unrecognized therapeutic or adverse effect of intervention.

Added to this list for readers and potential authors of *Journal of Conservative Dentistry*, with examples that our Journal has published in recent issues, might be:

- Demonstration in a patient of a phenomenon or response to an intervention using a newer material or technique that was previously demonstrated only in animal models, e.g.: pulpal response to a newer pulp capping agent.[17]
- Documentation of a new manifestation or finding, or clearer demonstration of a known feature of a disease, using a new technology or method, e.g.: using computed tomography for diagnosis of an unusual feature of a disease.[18]
- Documentation with clinical follow-up of a newer treatment method or intervention for a disease process, e.g.: a newer method of surgical decompression of an infected radicular cyst.[19]
- Demonstration of a previously unreported unique variation in root canal anatomical configuration, e.g.: a case report on the unusual location of second mesiobuccal canal orifice.[20]
- Demonstration of the efficacy of a newer technique of intervention with a sufficient period of follow-up to support, e.g.: using a newer material for furcal perforation repair with a sufficient clinical follow-up.[21]
- A previously unreported finding in a rare condition that suggests a possible pathogenetic mechanism.
- A new manifestation or finding, or clearer demonstration of a known feature of a disease, using a new technology or method.
- Demonstration, by means of modern technology, of known physiologic principles through the findings in a patient with a rare condition.
- A clinically important hazard or potential problem associated with the use of a diagnostic or therapeutic device or material.

## WHAT DOES AN EDITORIAL OFFICE LOOK FOR IN A SUBMISSION?

### Content of case reports

Sorinola *et al*.[22] surveyed the current advice available to authors of case reports from “instructions to authors” pages of a core collection of 249 journals (“Hague” list). These were examined and compared for advice or recommendation on writing case reports. The majority of information provided on the kind of case reports the journal publishes were on:

- whether the case has to be unusual or not was required by 99 (60%) of the surveyed journals,
- whether an instructive or teaching point was conveyed in the case report was required by 91 (55%) journals,
- whether the case is an original and innovative one was required by 42 (26%) journals, and
- Only 9 (6%) journals considered the hypothesis generation a reason for reporting the case.

### Instructions for authors

Journal editors differ in what they look for in a submission, but a review of the instructions to authors in 163 medical journals revealed a median limit of 1000 words, eight references, and six authors for case reports.[22] Approximately, 90% of the journals requested an abstract and key words. In this era of electronic databases, it is essential that you provide this information if you want your report to be accessible to the reader. Sixty-one percent were looking for the unusual or rare content, while 55% requested that the content should be instructive. In your cover letter to the editor, make sure that you sell your manuscript by articulating the salient educational message. Brevity and clarity are essential assets if a submission is to meet the journal’s space requirements and retain the reader’s interest.

### Manuscript review process

What happens with the case report once you submit it? First, the Editorial Office checks whether the manuscript meets the technical standards and that it is complete. If so, your manuscript moves to the next stage, and that is the editorial board meeting. Here we discuss your paper and judge whether it meets the standard of the Journal. This is a major hurdle, and we have to admit that not many manuscripts get beyond this stage. Next, we send your paper out for review, and after a receipt of the referee reports, one of the editorial board members issues a recommendation. The Editorial Board discusses the paper again, in view of the recommendation. If we agree that the case report is potentially interesting, we ask you to write a rebuttal and change the manuscript according to the issues raised by the reviewer. Now we have come to the final stage and here the editorial board member checks whether the referee’s issues have been dealt with. If there is any doubt at this stage, the paper can be rejected or we get back to you with additional questions. Finally, if you manage to get beyond this stage, your paper is accepted in the Journal and you can await publication.

Why do many case reports not get that far? As you may have noticed, we only publish two to three case reports each issue and with four Journal issues, it becomes clear that we cannot print all submissions. Indeed, we rejected 75 of the 86 case report submissions this year [Figure 1]. How do we decide what to take or not to take? We, the Editorial Board, are committed to the Journal, and we need to apply strict quality control measures in order to maintain the high standard of the journal.[23]

## HOW TO REPORT A CASE

The following section would deal on the essential basic nuances in the compilation of a case report. Case reports should be short and focused, with a limited number of figures and references. There are usually a restricted number of authors. The structure of a case report usually comprises a short unstructured (or no) abstract, brief (or no) introduction, report of the case, and discussion [Table 1]. Unlike original articles, case reports do not follow the standard IMRAD structure of the manuscript organization. As there is a wide variation in the format for case reports among different journals, it is essential for authors to follow exactly the target journal’s Instructions to Authors.

**Table 1:** Structure of a case report

Title
Authorship
Abstract
Introduction
Case report
Patient confidentiality
Figures
Tables
Discussion
Acknowledgements (optional)
References

### Structure of a case report

#### Title

The title should accurately and succinctly describe the case, and be sufficiently informative to interest the reader. Redundant words such as “case report” or “review of the literature” should be omitted. Clever or artistic titles should not be used because it is confusing and makes it difficult for the reader to determine the focus of the paper.

#### Authorship

Determining who will be listed as authors on a paper, and in what order, is an important process. It is convention that the author who does the most work on the project is listed first and only those involved in a substantive way are listed as authors. Past abuses in the authorship have created a need for clear authorship criteria, which have been provided by the International Committee of Medical Journal Editors (ICMJE).[24] Since it is unlikely that a single case will be managed by a large team of providers, one would not expect to see more than a few authors on a case report. One paper titled “Does it take a village to write a Case Report?”[25] demonstrates that some have successfully used the case report as a means to enhance their curriculum vitae. One study has objectively demonstrated that case reports contain too many authors.[26]

According to the International Committee of Medical Journal Editors guidelines, one may only be considered an author only if he or she meets all of the following three criteria:

- He/she has provided substantial contributions to conception and design, or acquisition of data, or analysis and interpretation of data.
- He/she has drafted the article or revised it critically for important intellectual contents.
- He/she has given the final approval of the version to be published.[24]

Anyone who does not meet all the three criteria, but who has contributed to the paper, may be thanked for their contribution in the acknowledgements section of the manuscript.[25,26]

#### Abstract

For some journals, no abstract is needed for case reports. If required, the abstract should be unstructured, and provide enough essential information for other researchers doing a database search. Abstracts for case reports are generally shorter than for other categories of papers, and are typically 100 words or less in length.

#### Key words

Use terms found in the Index Medicus database, which are called medical subheadings (MeSH). MeSH can be found at the PubMed home page (http://www.ncbi.nlm.nih.gov/entrez/query.fcgi?db=mesh). A list of additional words that may be unique to the case or to the topic are also discussed.

#### Introduction

The introduction section must state clearly why the case report is worth publishing and reading, not only because a statement of rationale is intrinsically logical but also because busy clinicians are unwilling to read an article if they cannot anticipate its interest or relevance to them and their practice.

The acceptable case report makes a contribution by illustrating a useful new approach to the diagnosis or the management of a condition or by offering a new insight into the pathogenesis of a disease. The introduction section should also contain some evidence from the literature to substantiate the authors claim that the case is important.

#### Case report

In writing a case report, the order of events should be presented in chronological order, typically comprising clinical history, physical examination findings, investigative results, differential diagnosis, working diagnosis, management, follow-up, and final diagnosis. Clarity is essential, especially with regard to important findings, all of which should be reported honestly. The presenting signs and symptoms should be objectively described, together with the relevant past dental history.

#### Patient confidentiality

Authors of case reports must be cognizant of the need to protect patient confidentiality, and specifically to safeguard protected health information, as defined by the Health Information Portability and Accountability Act (HIPAA).[27,28] The latter is defined by HIPAA as “any information that is entered, created, or received by health care providers that relates to the past, present, or future physical or mental health of any individual or to the provision of health care to that individual and that identifies the individual.”[28]

Preserving patient confidentiality is paramount. It is important that the patient is not identifiable from the information contained in the text of the case report. In the accompanying images, authors should make every effort to remove or conceal all identifiable features, taking particular care with the head and face. The eyes should be blanked out, and any birthmarks or tattoos concealed. It is preferable to obtain a written informed consent from the patient or parent/guardian (if the patient is a minor) giving permission to publish the case report and accompanying images.

#### Discussion

The discussion section serves to explain, clarify, and interpret key findings, and should be brief and to-the-point. An overview of the typical management may be required. The authors may suggest or explain their hypothesis, and express their own opinion here. A commentary that puts the case in context of other similar cases or explains specific management decisions is useful. Any shortfalls or limitations of the case should be stated. The value that the case adds to the current literature should be highlighted, so should differences between the reported case and other similar cases. Authors should also try to indicate the direction for future investigation, or the diagnosis or management of similar cases. In the last paragraph, the main conclusions of the case report, and an explanation of its importance or relevance should be provided. The take-home points should be emphasized, with focus on the main learning points, which should relate to the purpose for reporting the case.

A case report check list [Table 2] is being provided for future authors to use as a form of self-evaluation prior to submitting a manuscript to a journal to determine if further work is necessary before submission.

**Table 2:** Case report check list

General
Does the diagnosis satisfy accepted criteria, and are sufficient data provided to assure this?
If the report emphasizes a new observation, manifestation, intervention, or outcome, is sufficient information provided to convince the reader that it has not previously been reported?
Is a convincing case made that the features or events described were actually due to the condition or intervention under discussion?
Is enough detail provided so that the reader would be able to recognize and diagnose a similar case?
Is the case objective and devoid of unsubstantiated claims?
Is the case been clearly presented?
Has the case been prepared in accordance with the journal’s instructions for authors?
Is the length of the case report 1000–2500 words or less than the maximum allowed by the journal?
Title
Is it clear and easy to understand?
Does it accurately represent the report’s contents and focus?
Authorship
Do all authors meet the ICMJE criteria for authorship?
Are the authors listed in the order of contribution to the paper?
Are a reasonable number of authors been listed?
Abstract
Does it provide an accurate capsule of what was unique or especially instructive about the case and why it was published?
Does it agree completely with the body of the text?
Is everything in the abstract included in the report itself?
Has indexing terms from PubMed medical subheadings been provided as key words?
Introduction
Does it indicate specifically what the report is about and why it is important?
Does it adequately define and describe the entities to be discussed?
Does it make the clinical context of the report clear?
Are all acronyms or new terms defined?
Is it focused and concise?
Case Description
Has the case been described in a concise and clear manner?
Does it provide a clear picture of the patient’s presentation and condition?
If the report describes new, experimental, or unapproved interventions, is there evidence of appropriate institutional notification and approval?
Are the events and findings described in strict chronological order?
Are all the abnormalities described in the case description explained?
Has the salient aspects of the patient’s health history been clearly described?
Are the positive results and significant negative results pertinent to the examination been concisely presented?
Has the treatment procedures been clearly and concisely presented?
Patient Privacy
Has all identifying information been removed from the case report materials?
Has consent from the patient to publish the case and/or approval from Institutional Review Board been obtained?
Tables
Are the tables clear and easy to understand?
Is all the information included in the tables necessary?
Do the tables have a corresponding title?
Are the tables self-contained, needing no text to support them?
Figures
Are the included images the ones best suited to the case, rather than what just happened to be available?
Do the figures add to the report rather than duplicating information in the text or tables?
Are the figures of optimal magnification, resolution, and/or contrast, and appropriately cropped, for communicating what is intended?
Are the findings in the images made clear to the reader, including the use of arrows and labeling as appropriate?
Would inclusion of alternative or additional images have improved the clarity or teaching value of the report?
Do the legends make clear what the figures show?
Has written permission to publish photos of models or identifiable people been obtained?
Are the figures prepared according to the journal’s instructions to authors?
Discussion
Is there a clear statement of what is important about this case and why it was reported?
Has the case been compared to what is known in the literature?
Has the differential diagnoses been discussed?
Has the limitations of the case been offered?
Is everything in this section necessary and relevant to the case?
If the case is atypical or if there are unexpected features, are these explained?
If there are missing features or the included information is incomplete, is this acknowledged and explained?
Are the conclusions and recommendations made appropriately constrained and justifiable from the information presented?
Has the author avoided unwarranted extrapolation and generalization?
Does the discussion summarizes what the case contributes to the literature and states the overall conclusion learned from the study?
Conclusion
Does the conclusion relate to the purpose of the paper?
Has any new information learned from the case been summarized?
References
Does it appear that the author’s review of the literature was complete?
Are key original descriptions or essential prior reports included?
Are only directly relevant references included?
Are citations to obscure, outdated, or inaccessible sources avoided?
Are the references prepared as per the journal instructions for authors?

## ASSESSING THE VALIDITY AND EDUCATIONAL VALUE OF A CASE REPORT

Whether it documents new scientific knowledge or provides an educational resource on a previously known entity, the potential validity and value of a case report are determined by the following characteristics:

- How well the case is documented
- Its uniqueness and/or educational value
- The objectivity with which it is described
- How the information is interpreted with respect to broader principles and applicability to other patients

**Figure 2:**
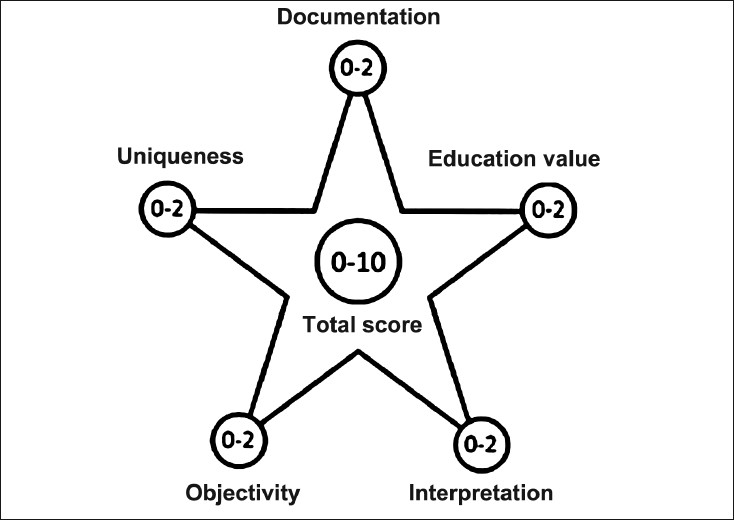
Piersons conceptual scheme for evaluating the quality of a case report

Pierson[29] has formulated a conceptual scheme with component domains for evaluating the quality of a case report [Figure 2 and Table 3].

**Table 3:** Evaluation of a case report according to the Piersons 5-component scheme

Component	Points	Criteria
Documentation	2	Complete, accurate, appropriate: everything needed to demonstrate that case is what the author contends it is, including appropriate diagnostic tests and images; no coexisting conditions or manifestations casting doubt on diagnosis, attribution of findings, or reasons for observed events; appropriate citation of references for case documentation
	1	Most usual criteria for diagnosis, manifestations, or outcome are fulfilled, although confirmatory or additionally instructive data/images not included; case is apparently as the author claims, although additional documentation would strengthen it; references appropriate but suboptimal
	0	Insufficient data provided to be certain that the reported findings, phenomena, or events were due to claimed mechanism and not something else; other explanations not adequately excluded; incomplete references for documentation
Uniqueness	2	Satisfactory demonstration that the manifestation, finding, complication, course, or intervention described has not previously been reported; appropriate citation of references to support uniqueness
	1	Although previously reported in the literature, this is the first report in this field or in this journal; references cited to substantiate this
	0	Subject of report has previously been documented in this field or in this journal; potential uniqueness cannot be determined from report
Educational value	2	Case described exactly fulfills accepted definition and/or description, without missing or atypical features; case is sufficiently “classic” or typical that reader could use it as a template for the future with respect to the condition or point under discussion; case and discussion facilitate comprehension and appreciation of topic; references complete, appropriately recent, and accessible, providing opportunity for further learning on topic
	1	Case has general attributes of claimed entity or occurrence, but with missing, atypical, or contradictory features rendering it less than a “classic” example; incomplete discussion of topic for optimal instructional benefit in allotted space; references less than ideal
	0	Case is sufficiently incomplete or atypical that generalization to other cases could be confusing or misleading; case lacks important aspects of “classic” description of entity under discussion; instructional content weak or very incomplete; references incomplete, irrelevant, outdated, or inaccessible
Objectivity	2	Data complete, contemporaneous, and presented in format appropriate for setting; no evidence of selective data presentation or emphasis; absent or atypical features identified and explained; possible alternative diagnoses or explanations listed and discussed; citation of alternative or contradictory sources provided if warranted; no evidence of author advocacy or bias related to conflict of interest
	1	Data presented in appropriate format but with uncertain completeness, timing or selection; evidence of subjectivity or selectivity in presentation of case; discussion presented such that incomplete or atypical features or alternative explanations are omitted or deemphasized; undue emphasis on references supporting author’s position
	0	Selective presentation of data; evidence of author bias in favor of claimed diagnosis, event, intervention, or commercial product, with insufficient presentation of inconsistent, or contradictory material; inadequate presentation and consideration of alternative explanations or approaches; only references supporting author’s position are cited
Interpretation	2	Conclusions and recommendations conservative, restricted to those consistent with and supported by evidence presented, and appropriately linked to cited literature; if reporting something new, acknowledgement by author of limitations of individual case and need for additional evidence; any conjectures about mechanisms or implications for therapy clearly identified as such; avoidance of general clinical recommendations extending beyond context of case
	1	Some conclusions overstep the data presented, although general clinical recommendations based on this case are avoided; incomplete linkage of presented data to literature
	0	Extrapolation of conclusions about mechanisms or interventions well beyond the data presented; literature citation in support of conclusions biased and/or incomplete; statement of general recommendations for patient management or use of therapy, clinical approach, or commercial product based solely on this case

Implications of total score: 9–10: report is likely to be a worthwhile contribution to the literature, 6–8: reader should be cautious about validity and clinical value of report, 5 or less: report is of insufficient quality for publication

The value of individual case reports should be kept in perspective in the larger context of the scientific literature. Appropriately viewed, however, the case report remains an important cog in the wheel of medical progress, which can stimulate clinicians’ interest, generate further research, or serve as a helpful educational tool. The application of the principles summarized in this article can help a potential author to prepare a case report in a more acceptable format and would help the reader to approach case reports critically and gain maximum benefit from them.

## SUMMARY

A case report will not have as much potential impact on the science or practice of health care as a randomized controlled trial or other research projects. However, it may be the only way to make others in the field aware of unusual presentations or complications, and it is a time-honored vehicle for teaching others. New syndromes, manifestations, associations, complications, or outcomes are appropriate subjects for case reports, as are typical and exceptionally well-documented examples of known entities that are relevant to a journal’s readers.

### Take-home points

1. The case reported should be unique, rare, or unusual
2. The manuscript should be short and succinct
3. The case should add a value to the diagnosis or the management
4. There should be a clear learning point.
